# An integrated approach using proximity labelling and chemical crosslinking to probe *in situ* host-virus protein–protein interactions

**DOI:** 10.1017/qrd.2024.19

**Published:** 2024-12-16

**Authors:** Jiaqi Li, Zhewang Lin

**Affiliations:** Department of Biological Sciences, National University of Singapore, Singapore

**Keywords:** dynamics, virology, protein–protein interactions

## Abstract

Host-virus interactions are critically important for various stages of the viral replication cycle. The reliance of viruses on the host factors for their entry, replication, and maturation processes can be exploited for the development of antiviral therapeutics. Thus, the identification and characterization of such viral-host dependency factors has been an attractive area of research to provide novel antiviral targets. Traditional proteomic efforts based on affinity purification of protein complexes from cell lysates are limited to detecting strong and stable interactions. In this perspective, we discuss the integration of two latest proteomic techniques, based on *in situ* proximity labelling and chemical crosslinking methods, to uncover host-virus protein–protein interactions in living cells.

## Introduction

**Figure 1.**
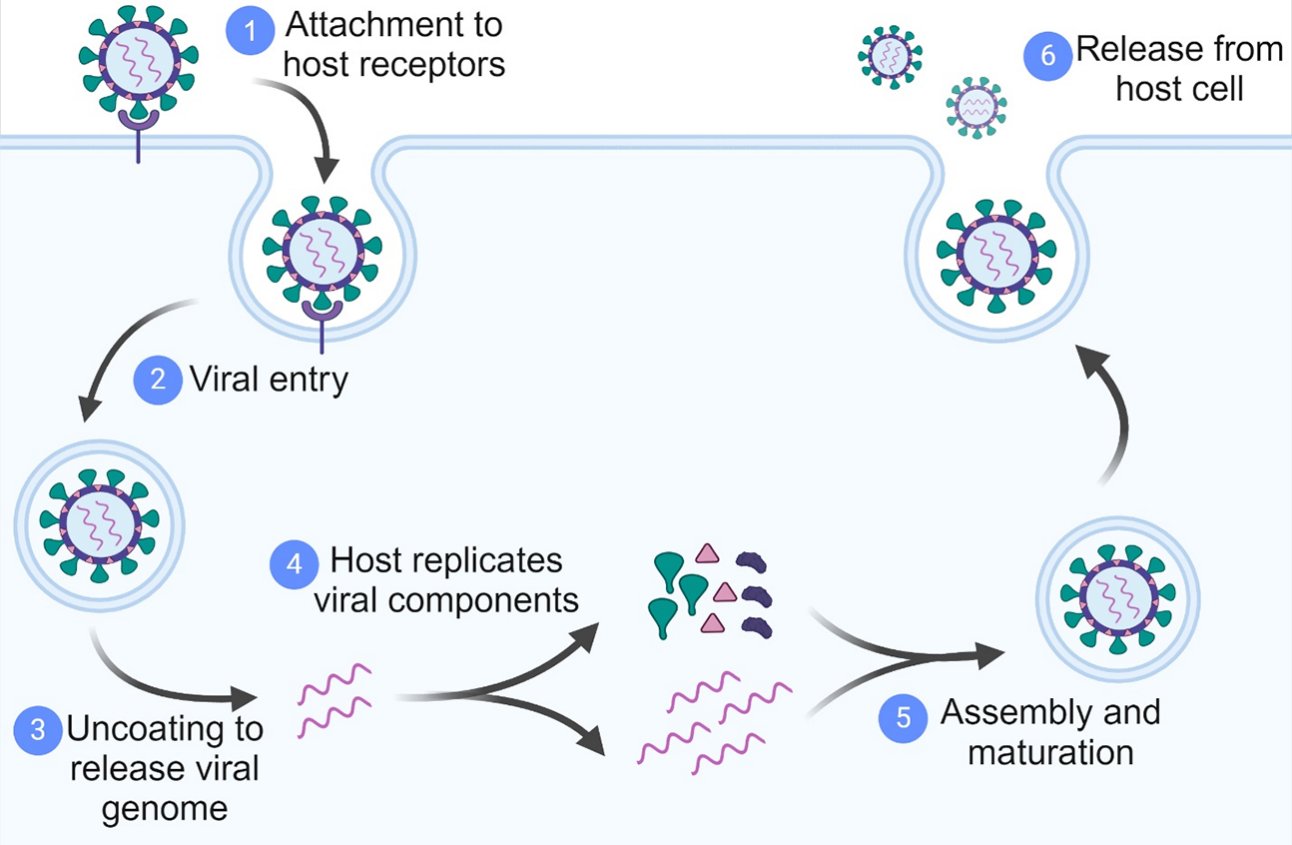
**A simplified life cycle of a virus**. The generic life cycle of a virus can be divided into six stages: 1. attachment of viral particles to host receptors on the cell surface; 2. entry of viral particles into the cell; 3. breakdown of capsid to release the viral genome; 4. expression and replication of the viral genome; 5. assembly and maturation of new viral particles from replicated viral components; 6. release of new viral particles from the host cell.

Viruses pose a constant threat to human health, yet effective antiviral treatments are not available for many viral pathogens, underscoring the need for novel antiviral targets. All viruses rely on the host and its cellular factors to complete various steps of their infection cycles (Figure 1). Therefore, one attractive antiviral strategy is to target and interfere with the host cell factors that are required by the pathogen for replication or persistence (Kaufmann et al., 2018). An ideal host factor target will be one that is non-essential for the host cell activity but a moderate inhibition of this factor will substantially impair virus production. Such a host-directed therapeutic approach is also less likely to have therapeutic resistance because resistance would require the virus to use an alternative host factor for replication. Thus, the identification and characterization of host-virus protein–protein interactions (PPIs) is an attractive area of research in virology. In addition to providing mechanistic insights into the viral entry, replication, and assembly processes, such studies can potentially identify novel host targets for the development of antivirals.

Traditional proteomic methods, such as affinity purification coupled mass spectrometry (AP-MS), have been widely used to study host-virus PPIs (Gerold et al., 2016; Lum and Cristea, 2016). AP-MS uses epitope tagging of the viral proteins or antibodies specific to the viral proteins for affinity purification of the viral protein “baits” and their associated proteins. Subsequent protein identification via mass spectrometry analysis provides a list of potential interacting proteins. While AP-MS has been successfully used to identify some host-virus PPIs, it also has its limitations. Firstly, the co-purification of interacting proteins with the bait relies on relatively strong and stable interactions. Thus, important but transient and weak host-virus PPIs may be missed. Secondly, cell lysis may dilute the protein concentrations and lose the physiological interactions. On the other hand, the mixing of cellular compartments during the lysis and purification process of AP-MS may also introduce false positive interactions.

In this perspective, we summarize recent advances in two different proteomic approaches to uncover *in situ* host-virus PPIs: proximity-based labelling methods and chemical crosslinking methods. Both methods are able to capture virus–host PPIs in living cells via covalent labelling of the interacting partners. However, these two *in situ* labelling methods still come with some drawbacks. Here, we discuss their strengths and limitations and the prospects of an integrated approach using these two complementary techniques to probe for host-virus PPIs in living cells.

### Principle of proximity labelling-based proteomic studies

**Figure 2.**
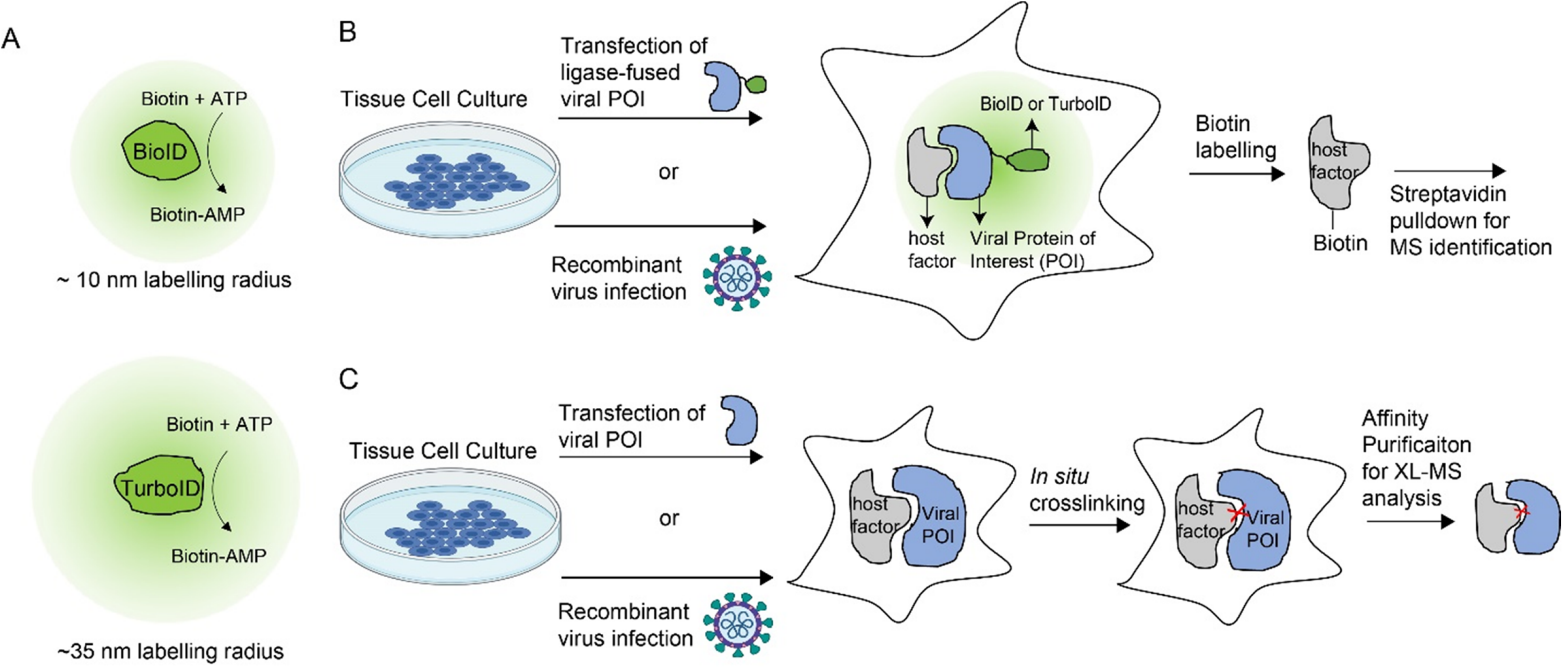
**Proximity labelling and chemical crosslinking methods to map host-virus PPIs**. A) Biotin ligase-based enzymes such as BioID and TurboID produce the biotin–5′-AMP as the reactive species for proximity labelling. The green clouds depict the labelling radius. B) General workflows for proximity labelling-based proteomics to identify host-virus PPIs. The biotin ligase-fused viral protein of interest can be introduced to cultured host cells by direct transfection as a single protein construct or infection as a replicating recombinant virus. *In situ* biotin labelling marks host interacting factors with a biotin label for subsequent pulldown and protein identification. C) General workflows of XL-MS to map host-virus PPIs. Viral proteins of interest can be introduced to host cells via direct transfection or recombinant virus infection. Host-virus interactions are captured by *in situ* protein–protein crosslinkers to enable subsequent affinity purification of viral proteins and XL-MS analysis.

Proximity labelling was developed as an alternative proteomic approach to map PPIs in living cells (Cho et al., 2020; Qin et al., 2021). This method involves the genetic fusion of the bait with promiscuous enzymes that convert inert small-molecule substrates into diffusible reactive species. Proximity-dependent labelling of interacting partners by the reactive species provides an “interactome history” of the bait in living cells and a molecular handle to isolate the interacting proteins for identification by MS analysis. Two main types of proximity labelling enzymes and probes were engineered: the peroxidase-based enzymes (APEX/APEX2) that use H_2_O_2_ as a co-substrate to oxidize the biotin–phenol substrate into a highly reactive phenoxyl radical; the biotin ligase-based enzymes (BioID/TurboID) that adenylates biotin using cytosolic ATP to form the reactive biotin–adenosine monophosphate (biotin–5′-AMP) intermediate (Figure 2A).

The activity and labelling kinetics of different proximity labelling enzymes have been extensively compared and summarized in other reviews (Cho et al., 2020; Qin et al., 2021). APEX2, the peroxidase-based approach, enables a high temporal resolution due to rapid labelling kinetics of less than 1 minute. However, the low cell permeability of the biotin–phenol substrate and the potential oxidative stress caused by the co-substrate H_2_O_2_ hinder the application of APEX2 to probe for host-virus PPIs in living cells. To our knowledge, no studies of host-virus interactions using APEX/APEX2 have been reported to date. In contrast, BioID and TurboID use the non-toxic and highly-soluble biotin substrate to initiate labelling, which is ideal for *in vivo* proximity labelling applications. BioID (Roux et al., 2012) is the first application of a promiscuous mutant of the *Escherichia coli* biotin ligase BirA (Choi-Rhee et al., 2004) that requires a long labelling time (>18 hours) due to its low enzymatic activity. More recently, the directed evolution of the biotin ligase BirA has led to the development of TurboID (Branon et al., 2018) with much faster labelling kinetics (≤ 10 minutes). Although the use of proximity labelling methods in virology is relatively new, both BioID and TurboID have been used to identify virus–host PPIs for a broad spectrum of viruses (Table 1). These were done either by the plasmid-encoded expression of the ligase-fused viral protein of interest in cells or by the generation of viruses expressing the fusion protein (Figure 2B). Here, we highlight a few recent examples of proximity labelling-enabled discovery of host targets for the development of novel antivirals.

**Table 1.** **Summary of the novel host-virus protein–protein interactions identified by proximity labelling methods**. “Biotin ligase” specifies the ligase used for proximity labelling. “Ligase Integration” indicates whether the ligase-fused viral protein of interest was expressed as a single protein or integrated in the context of a replicating recombinant virus. “Viral protein (bait)” indicates the viral protein of interest that was fused to the biotin ligase. “Identified interactors” lists the total number of candidate host interactors identified by the proximity labelling-based proteomic studies. “Validated novel interactors” lists the proteins that were validated by biochemical and functional studies. The proviral or antiviral roles of the validated host factors are indicated.

					Validated novel interactors				
Biotin Ligase	Ligase Integration	Virus*	Viral protein (bait)	Identified interactors	Protein	Uniprot ID	PDB ID	Role	References
BioID	Single protein	HIV–1	Gag	50	–	–	–	–	Ritchie et al. (2015)
BioID	Single protein	HIV–1	Gag	47	DDX17	Q92841	6UV0	Proviral	Le Sage et al. (2015)
					RPS6	P62753	5AJ0	Proviral	
BioID	Single protein	ZIKV	10 viral proteins	1224	–	–	–	–	Coyaud et al. (2018)
BioID	Single protein	EBV	LMP	1179	CD63	P08962	–	Antiviral	Rider et al. (2018)
					STAT3	P40763	6TLC	–	
					TSG101	Q99816	1KPP	–	
					HSC70	P11142	3FZF	Proviral	
					ITGB1	P05556	4WK4	–	
					Syntenin–1	O00560	1 N99	Proviral	
BioID	Single protein	IAV	PA-X	156	NUDT21	O43809	3BAP	Proviral	Gaucherand et al. (2019)
					CPSF6	Q16630	3Q2S	Proviral	
BioID	Single protein	HSV–1	glycoprotein M	170	–	–	–	–	Boruchowicz et al. (2020)
BioID	Single protein	HBoV1	nuclear protein 1	300	DHX15	O43143	5XDR	–	Wang et al. (2020)
					CPSF6	Q16630	3Q2S	Proviral	
BioID	Single protein	HIV–1	Vpr	352	APC1	Q9H1A4	4UI9	Proviral	Barbosa et al. (2021)
TurboID	Single protein	SARS-CoV–2	29 viral proteins	1388	ITGB1	P05556	4WK4	Proviral	Zhang et al. (2022)
					MAVS	Q7Z434	2MS7	Antiviral	
					SETD2	Q9BYW2	4FMU	Antiviral	
TurboID	Single protein	KSHV	IRF–1, IRF–4	213, 70	–	–	–	–	Kumar et al. (2021)
BioID	Single protein	CPV	NS2	122	–	–	–	–	Mattola et al. (2022)
BioID	Recombinant virus	RSV	NS1	271	MED25	Q71SY5	7EMF	Antiviral	Van Royen et al. (2022)
BioID2	Single protein	SARS-CoV–2	26 viral proteins	3011	–	–	–	–	May et al. (2022)
TurboID	Single protein	LASV	LASV polymerase	42	RARS	P54136	4R3Z	–	Fang et al. (2022)
					AIMP2	Q13155	5A5H	–	
					RPS3	P23396	5AJ0	–	
					PSMC5	P62195	5GJR	Antiviral	
					EIF4G2	P78344	4IUL	Antiviral	
					UPF1	Q92900	2GJK	Antiviral	
					GSPT1	P15170	5LZT	Proviral	
TurboID	Single protein	HCMV	US28	1,054	PDZ-RhoGEF	O15085	1HTJ	Proviral	Medica et al. (2023)
					p115-RhoGEF	Q92888	1IAP	Proviral	
					ROCK1	Q13464	2ESM	Proviral	
TurboID	Recombinant virus	HCMV	UL26	67	PIAS1	O75925	1 V66	Proviral	Ciesla et al. (2024)

* This table includes only animal viruses that have been studied by proximity labelling methods.

### Applications of proximity labelling methods in virology

Lassa virus, the cause of Lassa hemorrhagic fever, is highly prevalent in western Africa with an estimated death rate of 5000 per year (Hansen et al., 2021). Replication of Lassa virus in host cells critically depends on the virally encoded RNA polymerase but cellular contribution to these processes remained unclear. In 2022, Fang et al. generated a Lassa virus polymerase-TurboID fusion protein, verified that the fusion protein retained the polymerase activity, and performed TurboID-enabled proteomic analysis to define the Lassa virus Polymerase Interactome. 42 high-confidence Lassa virus polymerase interactors were initially identified (Fang et al., 2022). A functional screening using siRNA targeting each of the 42 high-confidence hits was performed to investigate the effect of gene knockdown via RNA interference (RNAi) on viral infection. The top hits from the RNAi screen revealed six antiviral host factors and one proviral host factor (G1-to-S-phase transition 1 (GSPT1) / eukaryotic peptide chain release factor (eRF3a)). Fang et al. further demonstrated that GSPT1 physically associates with Lassa virus polymerase. Pharmacological inhibition of GSPT1 via E3 ubiquitin ligase modulator induced GSPT1 degradation effectively inhibited Lassa virus growth in Huh7 cells. Although the exact mechanism and the functional consequence of the physical association between GSPT1 and Lassa virus polymerase remains to be clarified, this study demonstrates the potential of using proximity labelling-based proteomics to identify and characterize novel host-virus PPIs for antiviral developments.

A more recent example came from the application of TurboID to study host-virus PPIs in Human Cytomegalovirus (HCMV) (Ciesla et al., 2024). The HCMV protein, UL26, is important for high titer viral replications by preventing antiviral gene expressions, but the mechanisms involved are unclear. Ciesla et al. sought to identify host proteins that interact with UL26 during viral infection by a genetic fusion of TurboID to UL26 in the viral genome. Because UL26’s C-terminus is known to be critical for its function, the authors also generated a recombinant HCMV strain that expressed the TurboID tagged UL26ΔC variant as a control, reasoning that the proteins interacting with wild type (WT) UL26, but not the UL26ΔC mutant, are more likely to be important for UL26’s roles in HCMV infection. This well-controlled proximity labelling-based proteomic study identified 67 host proteins that preferentially interacted with the WT UL26. Many of the hits are STAT and PIAS family members which are involved in innate immune signaling, consistent with UL26’s role in modulating cellular antiviral response. Ciesla et al. validated that PIAS1 interacted with WT UL26 but not UL26ΔC. Most importantly, PIAS1 inactivation attenuated WT UL26 HCMV infection, resulting in an antiviral transcriptional environment similar to ΔUL26 infection. This TurboID-driven study uncovered a vital and potentially druggable UL26-PIAS1 interaction in modulating intrinsic antiviral defence during HCMV infection.

### Caveats of proximity labelling methods in the identification of host-virus PPIs

Despite the potential of proximity labelling methods in virology, there are some caveats of the methods that warrant considerations for the reliable identification of host-virus PPIs.

Tagging of the proximity labelling ligases to the viral protein could affect its physiological function. The addition of the 35-kDa BioID or TurboID to the viral protein might incur changes to the interactome profile of the viral protein of interest and affect its functions. For example, the fusing of TurboID to the envelop protein of the *Andrias davidianus* ranavirus (ADRV) was found to attenuate the viral infection due to a reduced virus adsorption efficiency (Jiang et al., 2023). Although slightly smaller versions of biotin ligases, such as the 27-kDa BioID2 (Kim et al., 2016) and 28-kDa miniTurbo (Branon et al., 2018), have been developed, tagging of these ligases remains a relevant concern. Furthermore, the location of the ligase tags on the viral protein could be important too. In 2015, two early applications of BioID to identify protein interactors of the HIV-1 Gag polyprotein were reported (Le Sage et al., 2015; Ritchie et al., 2015). During viral maturation, the HIV-1 Gag polyprotein is cleaved by the viral protease to the matured products: matrix, capsid, nucleocapsid, and p6. Ritchie et al. inserted the BioID ligase BirA* between the matrix and capsid in the Gag polyprotein, 12 amino acids upstream of the matrix-capsid cleavage site. They confirmed the ability of the matrix-BirA*-capsid construct to assemble and release virus particles and identified 50 cellular proteins as potential Gag interactors. Le Sage et al. constructed a Myc-BirA*-Gag fusion (the ligase was placed at the N-terminus of the matrix in the Gag polyprotein) and found 47 cellular interactors, among which they validated DDX17 and RPS6 via co-immunoprecipitation and western blot. Strikingly, there was only one overlap between the two studies: the protein lyrics which had been previously reported to interact with HIV-1 Gag. The results of these two early studies highlight the importance of ligase positioning in defining the biotinylation cloud surrounding the fusion protein and affecting the pool of identified interacting proteins.

False positives in the proximity labelling-based proteomics may inflate the list of identified interactors. In proximity labelling, the potential interacting proteins are biotinylated in a proximity-dependent manner and the biotin label is used as an affinity handle for enrichment via streptavidin-conjugated beads (Figure 2B). Thus, false positive hits in proximity labelling-based proteomics can arise from endogenously biotinylated proteins, nonspecific labeling or imperfect subcellular localization. Expression of the BirA* by itself is often used as a control to filter out false positive hits from the background labelling of BirA*. However, the subcellular location of the fusion protein might be different from the BirA* alone, leading to a different background noise. Perhaps the better controls to account for the nonspecific labelling are BirA*-tagged mutants that lack the WT viral protein function. In this way, interactors preferentially identified with the WT viral protein fusion in comparison to their corresponding mutant constructs are more likely to be important for the viral protein’s function.

Proximity labelling-identified proteins may not be the direct interactors of the viral protein bait. BioID and TurboID have an estimated labelling radius of ~10 nm (Kim et al., 2014) or ≥ 35 nm (May et al., 2020) respectively (Figure 2A), a parameter that may also vary with the labelling time. Thus, any proteins within the biotinylation cloud can be labelled and identified but they do not necessarily have direct interactions with the protein of interest. Together with the potential false positives from background noise, these caveats of proximity labelling-based proteomics necessitate follow-up experiments to verify the physical interactions and the functional relevance of the identified proteins. However, binary validations of direct interactions via immunoprecipitation and functional characterizations via RNAi-mediated gene knockdown or CRISPR-mediated gene knockout might not be practical for an inflated interactome list. Therefore, complementary *in situ* proteomic approaches, such as chemical crosslinking methods to capture direct interactions, might help narrow down the list of host-virus PPIs for subsequent functional studies.

### Chemical crosslinking proteomics as an orthogonal and complementary method for the identification of host-virus PPIs

Chemical crosslinking has been widely used to study *in situ* protein–protein interactions and provide structural insights on protein conformations (Graziadei and Rappsilber, 2022). This method generally relies on the use of cell-permeable protein–protein crosslinkers that can form covalent bonds between the amine (lysine residues) or sulfhydryl (cysteine) groups from two adjacent proteins. The covalent joining of the interacting partners in living cells captures transient PPIs and allows subsequent affinity purification under stringent denaturing conditions. This, in turn, helps to reduce nonspecific background during sample purification for MS analysis and minimize the false positives in identified interactions. Furthermore, crosslinking mass spectrometry (XL-MS) analysis reveals not only the identities of the interacting proteins, but also the corresponding crosslinked residues of the proteins, thereby confirming direct interactions between the identified protein pairs. With a defined length of the spacer arms of the protein crosslinkers, XL-MS analysis can also provide spatial information in the form of distance constraints between the crosslinked residues. More recently, a variety of enrichable, MS-cleavable, cell-permeable protein crosslinkers have been developed to reduce the computational search space of XL-MS and facilitate the proteome-wide discovery of crosslinked peptides (Kaake et al. 2014; Liu et al., 2015; Tang and Bruce, 2010).

A general workflow for targeted XL-MS to identify *in situ* host-virus PPIs includes the following steps: i) transfection of a plasmid encoding a viral protein of interest or infection of cells with viruses; ii) *in situ* crosslinking with a cell-permeable crosslinker; iii) quenching of crosslinking and lysis of cells; iv) affinity purification of the protein of interest and its crosslinked products for XL-MS analysis (Figure 2C). As an example, *in situ* XL-MS was used to reveal the interactions and topology of three viral proteins (Nsp1, Nsp2 and nucleocapsid) from the severe acute respiratory syndrome coronavirus 2 (SARS-CoV-2) (Slavin et al., 2021). In particular, two crosslinks between Nsp1 and the ribosomal subunit protein RPS3 and three crosslinks between Nsp1 and the eukaryotic translation initiation factor 3 (eIF3) were detected from the XL-MS analysis. These results were consistent with recent cryogenic electron microscopy structures showing that Nsp1’s C-terminal domain binds to and obstructs the mRNA entry tunnel and the roles of Nsp1 in mediating host translational shutoff (Schubert et al., 2020; Thoms et al., 2020; Yuan et al., 2020). Thus, the study by Salvin et al. demonstrated the use of *in situ* XL-MS to reveal direct interactions between viral and host proteins with topological information.

However, one obvious drawback of chemical crosslinking proteomics is that the covalent capture of an interacting protein pair relies on the presence of corresponding reactive amine or sulfhydryl groups at the binding interface. While the frequency of lysine residues in human proteins (~6% of all residues (Tekaia et al., 2002)) provides some coverage of lysine residues at the putative host protein binding face, it is important to check if the viral polypeptide chain contains lysine or cysteine residues to enable crosslinking.

### Outlook for integration of proximity labelling and chemical crosslinking methods to study host-virus PPIs

Proximity labelling and chemical crosslinking proteomics are powerful tools for studying transient PPIs in living cells. Recent years were marked by substantial advances in these methods: the development of TurboID with much faster labelling kinetics to improve the temporal resolution of proximity labelling studies and the development of MS-cleavable crosslinkers to enable proteome-wide *in situ* XL-MS analysis. We are just beginning to unleash the potential of proximity labelling and XL-MS in the field of virology. The advances in these techniques have now paved the way for an integrated proteomic approach to understanding the dynamic host-virus interactions during viral infections.

There are several synergies for these two *in situ* labelling methods to facilitate the discovery of novel host-virus PPIs. First, combining proximity labelling and crosslinking methods will likely help to narrow down the candidate list for follow-up studies. As for all methods involving MS analysis to identify prey proteins, the lists of interactors are often inflated by false positives due to various background noises. Overlapping the identified interactors from two orthogonal proteomics will help to provide a high-confidence list for subsequent functional validations. Secondly, while rapid proximity labelling by TurboID enables temporal recording of the host-virus interactome during the course of viral infection, XL-MS can be used to validate and distinguish direct and indirect interactions. Thirdly, XL-MS can be the alternative approach when the tagging of the biotin ligases to the viral protein of interest is not feasible due to the size of the ligases. This is because the purification of crosslinked products of viral protein of interest can be performed using antibodies against the target viral protein of interest or small affinity tags. Lastly, while proximity labelling-based proteomics is more likely to identify large interacting protein complexes, *in situ* XL-MS can be performed to provide topological information on these protein complexes during viral infection.

We expect to see more applications of these advanced proteomic techniques in virology in the near future. Identification and characterization of novel host-virus PPIs can improve our understanding of the essential molecular events needed for viral entry, replication, and maturation and provide new avenues for antiviral drug development.
