# Supplementary material for: An integrated approach using proximity labelling and chemical crosslinking to probe in situ host-virus protein–protein interactions
Source: QRB Discov. 2024 Dec 16;5:e11. doi: 10.1017/qrd.2024.19 (PMC11649372; doi:10.1017/qrd.2024.19)
Supplement: Peer review history

# Editor report: Recommendation: An integrated approach using proximity labelling and chemical crosslinking to probe *in situ* host-virus protein–protein interactions — R0/PR2

**DOI:** 10.1017/qrd.2024.19.pr2

**Affiliations:** Chemistry, Chalmers University of Technology, Sweden

No accompanying comment.

# Editor report: Decision: An integrated approach using proximity labelling and chemical crosslinking to probe *in situ* host-virus protein–protein interactions — R0/PR3

**DOI:** 10.1017/qrd.2024.19.pr3

**Affiliations:** Chemistry, Chalmers University of Technology, Sweden

No accompanying comment.

# Editor report: Recommendation: An integrated approach using proximity labelling and chemical crosslinking to probe *in situ* host-virus protein–protein interactions — R1/PR5

**DOI:** 10.1017/qrd.2024.19.pr5

**Affiliations:** Chemistry, Chalmers University of Technology, Sweden

No accompanying comment.

# Editor report: Decision: An integrated approach using proximity labelling and chemical crosslinking to probe *in situ* host-virus protein–protein interactions — R1/PR6

**DOI:** 10.1017/qrd.2024.19.pr6

**Affiliations:** Chemistry, Chalmers University of Technology, Sweden

No accompanying comment.
